# Effect of a Digital Health Physical Activity Program Integrating Gamification for Obesity Management in Comparison With Usual Care: Randomized Controlled Trial With an Ideographic Approach

**DOI:** 10.2196/78376

**Published:** 2025-11-28

**Authors:** Alexandre Mazéas, Aïna Chalabaev, Marine Blond, Charline Mourgues, Bruno Pereira, Martine Duclos

**Affiliations:** 1 Danish Centre for Motivation and Behaviour Science (DRIVEN), Department of Sports Science and Clinical Biomechanics, University of Southern Denmark, Moseskovvej 39, Odense, 5230, Denmark, 45 65504525; 2 SENS (Laboratoire Sport et Environnement Social), Université Grenoble Alpes, Saint-Martin d'Hères, France; 3 Kiplin, Nantes, France; 4 Department of Biostatistics unit, University Hospital Clermont-Ferrand, Hospital G. Montpied, Clermont-Ferrand, France; 5 Department of Sport Medicine and Functional Explorations, University Hospital Clermont-Ferrand, Hospital G. Montpied, Clermont-Ferrand, France

**Keywords:** behavior change, cost-benefit analysis, digital health, telemedicine, mHealth, gamification, intervention, physical activity, sports and exercise medicine, randomized controlled trial

## Abstract

**Background:**

Digital health interventions and gamification hold promise for managing chronic diseases, but evidence comparing their efficacy, long-term effectiveness, and cost-efficiency with those of usual care is limited. Moreover, there is a growing need for randomized controlled trials (RCTs) evaluating digital physical activity interventions to incorporate idiographic approaches and intensive longitudinal assessments that capture individual variability and the dynamic nature of behavior change.

**Objective:**

This 2-arm parallel RCT with embedded N-of-1 analyses examined whether a digital intervention integrating gamification and telecoaching (Kiplin program) outperformed a supervised, face-to-face, adapted physical activity program (usual care) in improving physical activity, clinical outcomes, and cost-effectiveness among adults with obesity and type 2 diabetes (T2D).

**Methods:**

We randomized (1:1) 50 patients with obesity or T2D (mean age 47.90, SD 12.49 years; 37/50, 74% female) referred to the University Hospital of Clermont-Ferrand, France, to either the Kiplin digital program or the usual care group. Both programs lasted 3 months, with a 6-month follow-up. The Kiplin intervention included 2 face-to-face and 20 online supervised sessions and 3 mobile app games, whereas the control group completed a standard hospital-based adapted physical activity (APA) program with 3 individual face-to-face, supervised sessions per week (36 sessions). The primary outcome was the change in daily step count, measured objectively and continuously via wearable devices from baseline to the end of the intervention. Secondary outcomes included changes in accelerometer-assessed physical activity, quality of life, body composition, physical capacities, and daily steps over 9 months. Program adherence was also evaluated. Mixed-effects models and generative additive models were conducted to analyze both between- and within-person evolutions in physical activity. A cost-utility analysis was computed to compare the cost-effectiveness of the programs.

**Results:**

Compared with usual care, Kiplin participants achieved greater increases in daily steps during both the 3-month intervention (+1085 steps/day) and follow-up (+1775 steps/day), with sustained effects over time. Idiographic analyses revealed marked heterogeneity, showing substantial between- and within-person variability, with 9 participants exhibiting nonlinear patterns and divergent individual trajectories, with some participants showing no improvement. No significant group differences were observed in secondary clinical outcomes, except for change in moderate-to-vigorous physical activity, in favor of the Kiplin group. Cost-utility analyses showed no significant difference between programs. Kiplin participants attended an average of 14.68 of 22 possible APA sessions and engaged in an average of 2.6 games. In contrast, usual care patients attended an average of 30.27 of 36 APA sessions.

**Conclusions:**

This study demonstrates the potential of digital gamified interventions to promote and sustain physical activity, offering an alternative to face-to-face programs. However, individual heterogeneity in the response to the intervention highlights the need for screening tools and tailored approaches. Further large-scale studies are warranted to evaluate the long-term clinical and economic impact of such interventions.

## Introduction

Adapted physical activity (APA) is an evidence-based, multidisciplinary rehabilitation program currently considered the gold standard treatment for obesity or type 2 diabetes (T2D) because of its multiple benefits such as weight and fat loss and improvements in blood pressure, cardiorespiratory fitness, insulin sensitivity, appetite control, and quality of life [1]. The economic impact of such programs could be substantial as, in France, the estimated health care savings per individual who becomes sustainably active amount to €840 (US $967.86) for individuals aged 20 years to 39 years and €23,275 (US $26,817.70) for individuals aged 40 years to 74 years [2].

However, despite these well-documented benefits, APA programs often struggle with low adherence and observance rates [3], with reported dropout rates reaching approximately 50% [4,5]. Moreover, only a low percentage of patients maintain their physical activity (PA) at the end of these programs [6-8]. For instance, a systematic review published in 2017 revealed that only 33% of participants adhered to prescribed exercise programs following the completion of supervised training [8]. Logistical and financial barriers further limit the impact of these programs, including factors such as inconvenient timing, transportation difficulties, program cost, and location [9,10]. Additional challenges such as lack of self-discipline or motivation, pain or physical discomfort, and lack of time are generally mentioned by patients with obesity [11].

Given these challenges, there is increasing interest in leveraging digital health technologies for obesity treatment. Digital health interventions hold promise to better change behavior in real-life contexts and have the potential to extend the reach of evidence-based behavioral interventions at lower cost while reducing patient burden [12]. Multiple interventional perspectives emerge with the democratization of digital tools. Among these innovations, gamification stands out; it is defined as the use of game mechanisms such as points, levels, leaderboards, badges, challenges, or customization in non-game contexts to foster behavior change, engagement, and motivation and to solicit participation. Recent meta-analyses suggest that gamified interventions are effective at promoting long-term changes in PA among various participants, including clinical populations [13-15]. Notably, compared with digital interventions that do not incorporate game mechanics, gamified interventions produce larger effects on PA (*g*=0.23) [14] and improvements in daily step count (+489 steps/day), BMI (−0.28 kg/m²), body weight (−0.70 kg), body fat percentage (−1.92%), and waist circumference (−1.16 cm) [15]. These findings suggest that gamified interventions not only promote behavioral change but also enhance the overall effectiveness of traditional digital health programs. In addition, telehealth solutions are emerging as a safe alternative for proposing remote APA sessions with good acceptability among patients and positive clinical results [16-18].

Nevertheless, despite the promise of digital health interventions, the evidence supporting their use in obesity treatment remains limited. Although digital interventions have been extensively tested in comparison with passive or active control groups, no rigorous trial has yet demonstrated the superiority of digital PA interventions over existing ones (eg, supervised and APA programs). In addition, evidence regarding long-term effects and cost-effectiveness remains inconclusive [19-21]. In this context, the digital health domain needs more rigorous evaluations, including follow-up assessments, comparisons with usual care, and economic evaluations.

In addition, although the gold standard evaluation method, namely the randomized controlled trial (RCT), can be helpful for evaluating the efficacy and effectiveness of digital health tools [22,23], evaluation processes must extend beyond the average response in order to consider the idiosyncratic nature of PA (ie, the fact that people’s activity and responses to an intervention can largely differ from one person to another) [24]. This emphasizes the need to adopt an idiographic approach (ie, individual statistical modeling) and report both between-group differences in benefit and within-person evolutions [25]. Finally, as the PA behavior change process is dynamic, it requires high-resolution measurements in order to capture its relative variability or stability over time [24,26]. Since intervention effects can be nonlinear and vary across time scales, the traditional measurement bursts conducted in PA trials (ie, 7-day measurement before and after the intervention) are not adapted to properly assess the impact of a behavioral change intervention on PA [24]. To answer these 2 challenges, integrating intensive longitudinal measures with N-of-1 methods within an RCT may prove particularly valuable [25].

Herein, we present the results of the Digital Intervention Promoting Physical Activity among Obese people (DIPPAO) study, an RCT designed to compare a digital intervention integrating gamification and telecoaching (the Kiplin program) with usual care. The study tests the hypothesis that these 2 interventional features can augment PA levels during and at the end of the program – ultimately leading to better clinical outcomes in the long haul. To address limitations observed in previous studies, this trial included (1) a 6-month follow-up to assess the sustainability of the intervention effect after the end of the intervention, (2) a cost-utility analysis to assess the economic impact of both programs, (3) an intensive longitudinal assessment of daily steps throughout the entire study to capture daily PA behavior more precisely, and (4) an analysis of both between-group differences and within-person evolutions using an idiographic approach.

## Methods

### Overview

The DIPPAO study is a 2-arm parallel prospective randomized controlled trial conducted in France. Full details of the study methods have been reported previously [27]. This trial aimed to compare the effectiveness, cost-effectiveness, and psychological mechanisms of the Kiplin program in comparison with face-to-face, supervised PA sessions among patients treated for obesity or T2D. The authors are solely responsible for the design and conduct of this study; all analyses, drafting, and editing of the paper; and its final contents. In this paper, we report the primary and secondary clinical outcomes along with the cost-utility analysis; the psychological mechanisms results are reported separately. There were no significant deviations from the prespecified protocol during the trial. The reporting follows the Consolidated Standards of Reporting Trials (CONSORT) guidelines [28] and the Template for Intervention Description and Replication checklist [29].

### Ethical Considerations

#### Ethics Approval

The protocol of this trial was reviewed and approved by the National Human Protection Committee (CPP Ile de France XI, number 21 004‐65219). All eligible individuals were informed about the study’s objectives, procedures, and ethical considerations, including assurances of confidentiality, and provided written informed consent prior to participation.

#### Compensation

Participants did not receive monetary compensation; however, the wearable device distributed at the beginning of the study was offered to them upon its completion.

#### Privacy and Confidentiality

Our trial was conducted in accordance with established standards for privacy and confidentiality. All investigators with direct data access took appropriate measures to safeguard the confidentiality of information related to the medical products, the trial, and the participants, particularly their identity and outcomes. No identifiable patient information is included in the manuscript, the supplementary materials, or the project’s Open Science Framework page.

### Study Population, Context, and Procedure

#### Context and Settings

The study was conducted within the department of sports medicine of the University Hospital of Clermont-Ferrand, France, located in an urban area with approximately 500,000 inhabitants. The hospital is situated within the city and is easily accessible by car and public transportation. The unit admits around 450 patients with various chronic diseases annually and is equipped with a dedicated clinical research office, electronic health record systems, and specialized facilities for supervised APA sessions. Participants were referred to the program by their general practitioners. The intervention programs were provided free of charge to participants; however, transportation to the hospital was at their own expense. Patients’ availability for participation was strongly influenced by their occupational commitments. In-person sessions were offered at multiple times throughout the week to maximize feasibility for the patient.

#### Participants

Patients were screened between June 2021 and October 2022. Eligible patients were 18 years to 65 years old who were treated for obesity (BMI ≥30 kg/m^2^ and <45 kg/m^2^) or overweight or obesity and T2D within the department of sports medicine. Patients were required to own either an Android-based phone or Apple iPhone with a study-supported operating system. Full inclusion and exclusion criteria are available in Table S1 in Multimedia Appendix 1. Participants attended 5 visits throughout the study: a selection visit, an inclusion visit, and 3 experimental visits.

#### Selection and Inclusion Visits

During the selection visit, one of the investigating physicians checked the patients’ eligibility criteria and signed the informed consent form. Only after providing informed consent did participants proceed to the inclusion visit, where they were given a wearable device (Garmin Vivofit 3; Garmin International) and an accelerometer (Actigraph GT3x; ActiGraph LLC) for baseline assessment of PA over a 7-day period. Randomization was performed by the associate biostatistician using a permuted block design with variable block sizes and a 1:1 allocation ratio. The randomization list was transmitted using sequentially numbered, opaque, sealed envelopes to the data collectors.

#### Experimental Visits

The T0 experimental visit marked the baseline assessment conducted prior to the start of the intervention. At the conclusion of the 3-month program, participants completed the T1 experimental visit. To evaluate the long-term effects of the intervention, the T2 experimental visit was conducted 6 months postprogram completion. Research assistants collecting data were blinded to treatment allocation. However, double blinding was not feasible in this context because the intervention’s nature made it impossible to conceal allocation from the participants.

### Intervention Overview

#### Intervention Group

**Figure 1.**
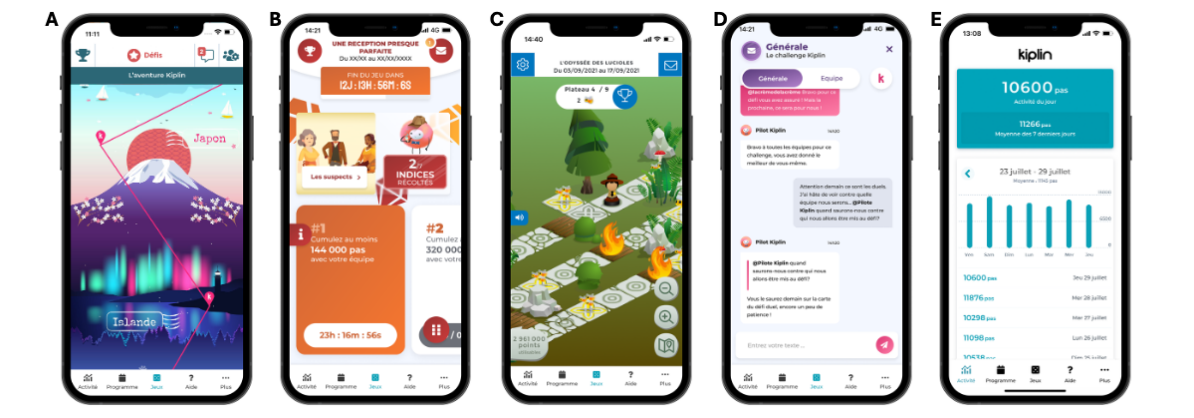
Screenshots of the Kiplin app: (A) The Adventure, (B) The Mission, (C) The Board Game, (D) the chat, (E) the activity monitoring tool.

Details on the intervention content have been reported previously [27,30]. The Kiplin intervention is a theory-driven digital APA intervention grounded in the social identity approach and self-determination theory. It comprises 4 key components: (1) 22 APA sessions (2 face-to-face sessions at the program’s start, followed by 20 remote videoconference sessions); (2) 3 PA collective games accessible through an Android or iOS app; (3) chat feature allowing communication with other participants, teammates, and health professionals; and (4) an activity monitoring tool enabling participants to track their PA in real time (Figure 1). The intervention integrates a total of 16 behavior change techniques and primarily aims to promote changes in daily PA behavior.

Participants completed 3 sessions per week during the first 2 weeks (1 face-to-face and 2 telecoaching sessions), 2 telecoaching sessions per week during the following 6 weeks, and 1 telecoaching session per week during the third month, for a total of 22 sessions. The 2 initial face-to-face sessions were conducted at the University Hospital under the same conditions as the control group (see the following paragraphs) and were designed to ensure that participants correctly adopted the required movements. The telecoaching sessions consisted of 60-minute, group-based live APA classes delivered remotely by a professional APA coach to small groups of 5 to 7 participants. These participants played the Kiplin games together then met for the group sessions, fostering both social connection and engagement. Each week, several session time slots were available, allowing participants to register according to their preferences and availability. Sessions incorporated interactive elements such as quizzes, riddles, and practical tips on PA, in addition to endurance, muscle strengthening, and stretching exercises, as well as therapeutic education.

Participants engaged in 3 distinct games, with no option for selection. In all games, participants’ daily step counts were converted into points, enabling progression within the game environment. The Kiplin app retrieved participants’ daily step counts via integration with the application programming interface of the tracking app (Garmin Health in this study). In The Adventure, the objective was to collectively achieve step goals to progress toward a final destination; players tracked their progress on a digital world map with checkpoints representing distances between cities (Figure 1A). In The Mission, participants completed PA and collective challenges to unlock clues and solve missions (Figure 1B). Finally, in The Board Game, participants acted as forest rangers tasked with extinguishing fires. Meeting step goals enabled progress on the board and advancement to higher levels, with the ultimate goal of extinguishing all fires and saving forest residents (Figure 1C).

These games incorporated multiple gamification mechanisms such as points, trophies, leaderboards, chat features, challenges, and narratives (see [30] for an overview of Kiplin’s gamification strategies following the taxonomy proposed by Schmidt-Kraepelin et al [31]). The telecoaching APA coaches also participated in the games alongside the participants and were available to answer game-related questions at the end of telecoaching sessions.

#### Control Group

Participants allocated to the control condition received the traditional 3-month face-to-face, supervised APA program at the University Hospital of Clermont-Ferrand, with 3 sessions a week on nonconsecutive days for a total of 36 sessions. The sessions were conducted individually, with no interaction between participants. Each session included a warm-up and 50 minutes of endurance and muscle-strengthening exercises, followed by stretching, all under the supervision of an APA coach in a dedicated facility. Aerobic and resistance exercises were organized in a circuit comprising 6 exercise stations (3 aerobic and 3 resistance). Aerobic exercises were performed at 50% of VO_2_max during the first week, with intensity progressively increased by 10% every 2 weeks to reach at least 80% of VO_2_max during the final 9 weeks. Resistance exercises were performed at 50% of 1-repetition maximum (1RM) in the first week, with the load similarly increased by 10% every 2 weeks to reach and maintain 80% of 1RM during the final 5 weeks.

### Study Outcomes

Full details of the outcome measures, including their reliability and validity, have been reported previously [27]. At baseline, participants provided demographic data, including date of birth, sex, and highest level of education completed. The primary outcome was the change in daily step count, assessed at high resolution using the Garmin Vivofit, a wearable activity tracker with validated accuracy under various walking conditions [32], from baseline to 1-week postintervention. Additionally, the change in daily steps in the follow-up period from baseline was also evaluated.

The 3 experimental visits included the following clinical outcomes. BMI was calculated as body mass (kg) divided by height squared (m²). Body composition, including fat and lean mass, was assessed using bioelectrical impedance analysis with the multifrequency segmented body composition analyzer (Tanita MC780; Tanita). Moderate-to-vigorous PA (MVPA), light PA (LPA), and sedentary time were measured over 7 consecutive days using a triaxial accelerometer (ActiGraph GT3x). Cardiorespiratory fitness was assessed via the 6-minute walk test (6MWT). Muscular strength of the upper limbs was assessed via handgrip strength using a dynamometer (Takei Grip-D; Takei) with a series of 3 handgrip test measurements for right and left hands in the seated position. Muscular strength of lower limbs was assessed through maximum knee extension torque using an isokinetic dynamometer at speeds of 30 °/s, 60 °/s, and 120 °/s. Quality of life was measured via the EQ-5D-5L questionnaire [33], which evaluates the following 5 dimensions: mobility, autonomy of the person, current activity, pain and discomfort, and anxiety and depression.

For both groups, the number of APA sessions attended was assessed. For the Kiplin group, app engagement was collected and comprised participation in the games, app usage frequency, and the number of messages sent.

The health economic evaluation was conducted through a cost-utility analysis incorporating the (1) identification and valuation of costs and (2) measurement of utility using the EQ-5D questionnaire. The analysis was performed from the health insurance perspective considering only direct medical costs. The time horizon for this evaluation extended from baseline (T0) to the follow-up assessment at T2.

### Statistical Analysis

The trial was designed to demonstrate a difference equivalent of an effect size of *d*=0.6 on our primary outcome (daily steps) for 80% power and a 2-sided type I error at 0.05. In idiographic designs, statistical experts [34] recommend a minimum of 835 observations for adequate power (.80) at level 1. With 2058 (98 days × 21 participants) observations, we had adequate power to detect within-person changes in the Kiplin condition [35].

All analyses followed a modified intention-to-treat principle, incorporating participants with complete data for the primary or secondary outcomes at the initial 2 experimental visits. This pragmatic analytical approach aimed to enhance internal validity by assessing treatment effects among participants who received the intervention while acknowledging that the necessary exclusions could attenuate some benefits of randomization. Analyses were performed using R (R Foundation for Statistical Computing). Baseline variables are reported as numbers and percentages for categorical variables and means with SDs for continuous variables. According to the CONSORT 2010 statement [28], group differences in baseline variables were not compared using significance testing. Two authors directly accessed and verified the underlying data reported in the manuscript, including one who had no affiliation with the Kiplin company.

Changes in daily step count were assessed between baseline and intervention periods as well as between baseline and follow-up using linear mixed-effects models. These models accounted for the nested data structure and included fixed effects for group, time, and group × time interaction. Because plots of the residuals and estimated trajectories suggested substantial nonlinearity in step count change over time, we compared polynomial extensions of the time effect (quadratic, cubic, and quartic) by minimum Akaike information criterion (AIC) and Bayesian information criterion (BIC). The quadratic model provided the best fit and is presented in the Results section. Random intercepts for participants and random linear slopes for repeated measures at the participant level were included. As an additional analysis, we treated measurement occasion as a categorical “period” factor (baseline, intervention, follow-up) rather than as a continuous measure of days. In this model, we included fixed effects for group, period, and the group × period interaction with the same random effects structure.

Adjusted models were further fitted and incorporated fixed effects for group, period, group × period interaction, age, baseline PA levels, BMI, season, and number of completed APA sessions – factors previously identified as significant predictors of intervention effects [30]. Nonwear days, defined as days with fewer than 1000 steps, were treated as missing data. All models were performed using the *lmerTest* package [36], and contrast analyses were conducted using the *emmeans* package [37]. Diamond comparison plots were drawn with the R package *ufs* [38].

In a complementary analysis of the primary outcome, an idiographic approach was used to analyze daily step counts for each participant separately using generalized additive models (GAMs) [39]. GAMs are an extension of generalized linear mixed models that allow the estimation of smoothly varying trends where the relationship between the covariates and the response is modeled using smooth functions [40]. GAMs are particularly well-suited to the modeling of time series data with 1 level of measurement (ie, repeated measurements nested within 1 individual), as they can accommodate the inclusion of autocorrelated error terms [41]. Nonlinearity was assessed via the effective degrees of freedom (edf) of smoothing terms, with edf ≥3 indicating nonlinearity [41]. GAMs were computed using the *mgcv* package [42], and the *visreg* package [43] was used for model visualization.

Continuous secondary outcomes were analyzed using mixed-effect models (including the group, time, and group × time interaction terms as fixed effects) to assess changes across 9 months (ie, considering the 3 measurement points). As statistical power was computed for the primary outcome, secondary outcome analyses were classified as exploratory [44].

## Results

### Participant Characteristics

**Figure 2.**
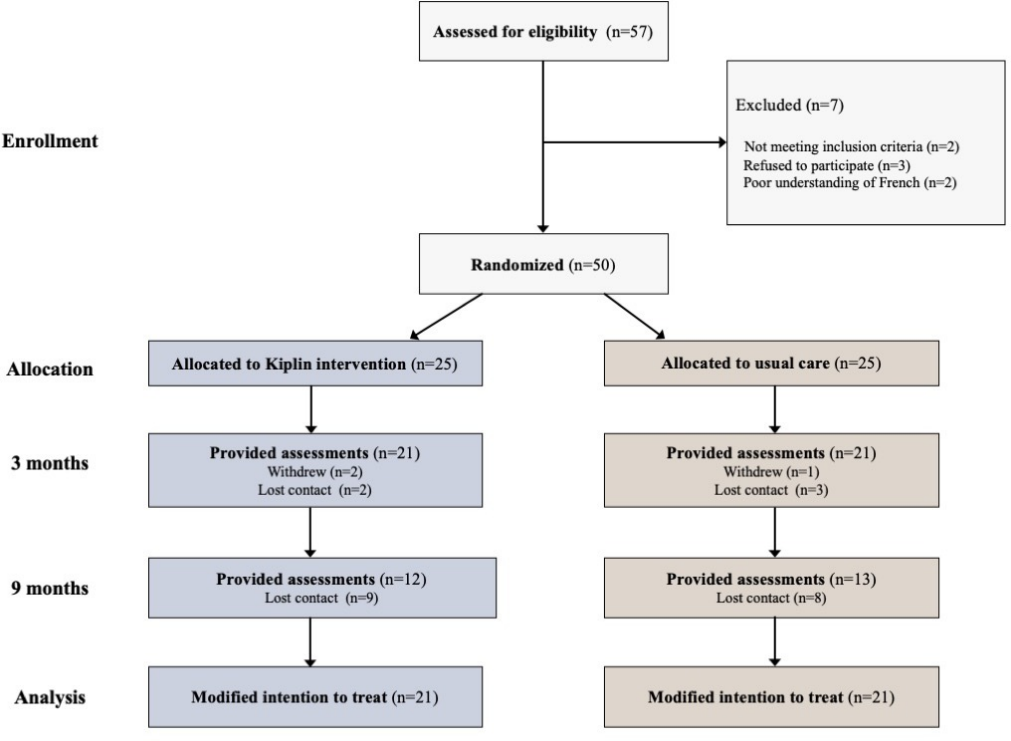
Study flow diagram.

Between June 22, 2021, and October 6, 2022, 57 patients were screened for eligibility, with 50 meeting the inclusion criteria and subsequently randomized to either the Kiplin group (n=25) or usual care group (n=25; Figure 2). Participants who completed at least 2 assessments (modified intention-to-treat sample) had a mean age of 47.7 (SD 13.3) years and a mean BMI of 40.0 (SD 7.14) kg/m², and 31 were women (31/42, 74%). A total of 13 participants had T2D (13/42, 31%). At baseline, the average daily step count was 6584 (SD 3787) steps per day. No substantial differences in baseline characteristics were observed between groups (Table 1). The descriptive statistics of all the randomized participants are available in Table S2 in Multimedia Appendix 1.

**Table 1.** Descriptive statistics of the modified intention-to-treat sample.

Characteristics	Kiplin intervention (n=21)	Usual care (n=21)	Total (n=42)
Sociodemographics			
Age (years), mean (SD)	47.5 (11.0)	47.89 (16.13)	47.7 (13.3)
Female, n (%)	17 (81)	14 (67)	31 (74)
BMI (kg/m²), mean (SD)	39.67 (7.37)	40.32 (7.02)	40.0 (7.14)
Obese, n (%)	20 (95)	19 (90)	39 (96)
T2D^a^, n (%)	7 (33)	8 (31)	13 (31)
Education			
Less than high school, n (%)	5 (24)	8 (38)	13 (31)
High school, n (%)	6 (28)	3 (14)	9 (21)
University degree (%)	10 (48)	10 (48)	20 (48)
Physical activity at baseline (daily steps), mean (SD)	6340 (3269)	6827 (4249)	6584 (3787)

a T2D: type 2 diabetes.

### Compliance and Engagement Metrics

Among the 50 randomized participants, 8 withdrew or were lost before program completion (3 in the intervention group and 5 in the control group), and 17 were lost during the follow-up period (Figure 1). Of a possible 274 days, participants logged steps for a median of 158 (IQR 162) days throughout the study, with 80% (34/42) of participants wearing their activity monitor for at least 75% of days. In the Kiplin condition, participants attended an average of 14.68 (SD 6.14) of 22 possible APA sessions, yielding a completion ratio of 0.67 (SD 0.28). They engaged in an average of 2.6 (SD 0.71) of a possible 3 PA games, corresponding to a mean of 36.6 (SD 10.02) days in-game; sent an average of 14.2 (SD 15.93) messages; and logged into the app on 88.12 (SD 43.63) times on average. In contrast, patients receiving usual care attended an average of 30.27 (SD 5.88) of 36 APA sessions, with a completion ratio of 0.84 (SD 0.16).

### Primary Outcome

#### Between-Group Differences

**Figure 3.**
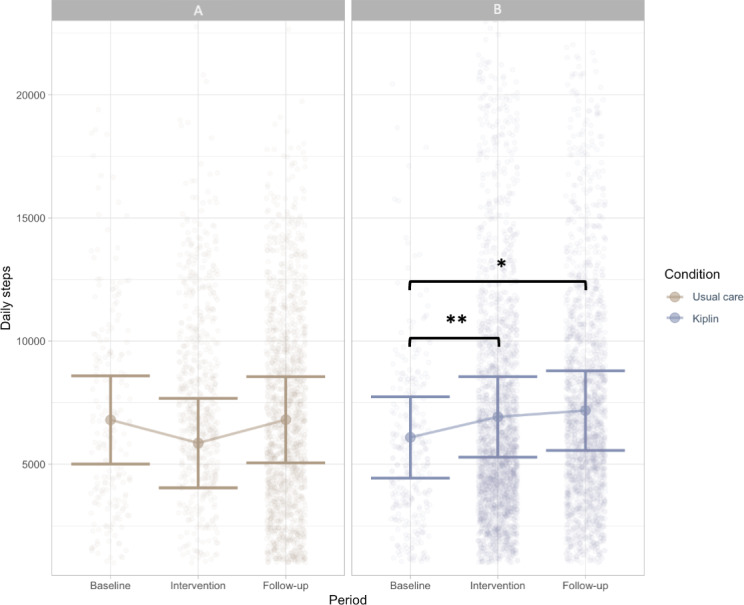
Changes in daily steps throughout the study phases for the (A) usual care and (B) Kiplin conditions, with the error bars representing the standard error. *P=.01; **P<.001.

During the 3-month intervention period, participants in the Kiplin condition increased their daily steps by 1092.06 (95% CI 533-1651.5) steps per day on average (+10.9%; *P*<.001; *d*=0.38) compared with the baseline period and by 834 (95% CI 114-1554.7) steps per day on average during the follow-up period (+8.3%; *P*=.01; *d*=0.29) compared with baseline. In contrast, the daily step count of the usual care condition remained stable and showed no significant changes in daily steps during the intervention period (+0.1%; *P*≥.99) or follow-up period (+0.9%; *P*=.10) relative to baseline. Figure 3 depicts the unadjusted daily step count evolution by phase and condition.

**Figure 4.**
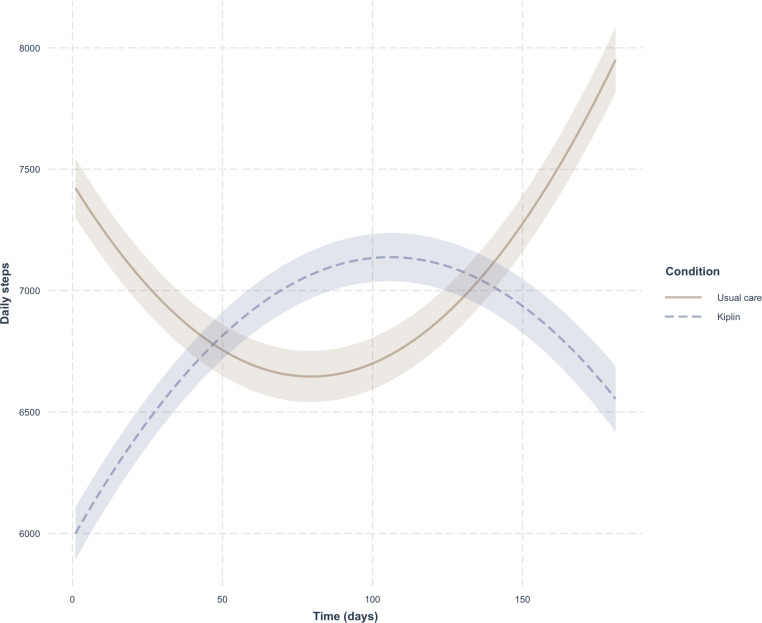
Quadratic growth curves of daily steps for the Kiplin and usual care conditions.

Results of our quadratic mixed-effects model predicting daily step count from time (days since baseline) indicated that the time trajectory of daily steps differed significantly between the Kiplin and usual care conditions (Figure 4). The usual care group exhibited a nonsignificant linear decline of 20.06 steps per day (*P*=.07), followed by a small but significant upward curvature (*ß*=0.126, *P*=.03). In contrast, the Kiplin group’s linear slope was 41.98 steps per day steeper than than that of the control (*P*=.004), and its quadratic term was 0.229 steps/day² lower (*P*=.005).

In our mixed-effects model predicting daily step count from period (baseline, intervention, follow-up), the contrast analyses revealed that participants of the Kiplin group had a significantly greater increase in mean daily steps between baseline and the intervention period compared with the usual care group, with an estimated difference of 1085 (95% CI 493-1676) steps (*P*<.001) relative to baseline. Similarly, during the follow-up period, the daily step count change from baseline for participants in the Kiplin group remained significantly greater, at 1775 (95% CI 906-2643) steps (*P*<.001).

Results of the adjusted models follow the same trends and are available in Tables S3 and S4 in Multimedia Appendix 1.

#### Within-Person Evolutions

**Figure 5.**
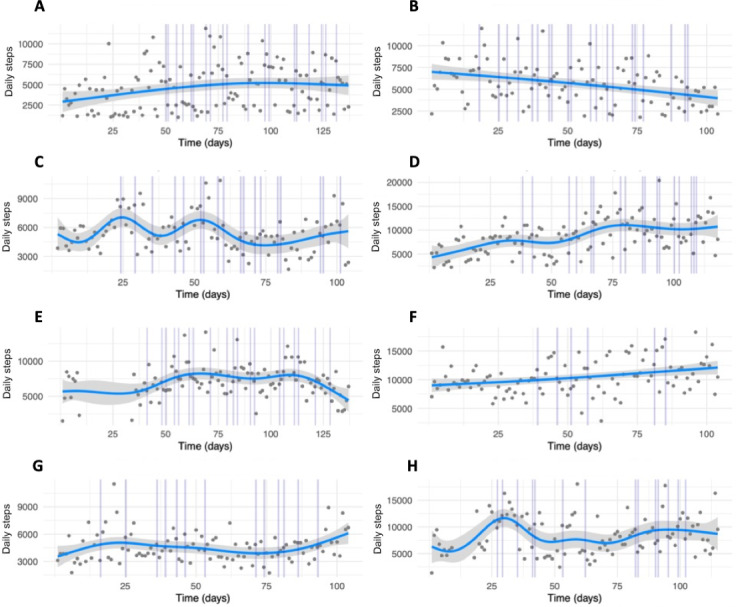
Plots of the generalized additive models for the evolution of daily steps from baseline to 1-week postintervention, with vertical lines representing adapted physical activity sessions attended, for the 8 participants of the Kiplin condition who had significant changes over time: (A) participant #2, (B) participant #12, (C) participant #7, (D) participant #15, (E) participant #8, (F) participant #19, (G) participant #9, (H) participant #21.

The results of the GAMs analyzing the evolution of daily step counts over time for each participant in the Kiplin group are summarized in Table S5 in Multimedia Appendix 1. The edf for the smoothing terms ranged from 1.0 to 8.10, with 9 participants exhibiting edf>3, indicative of nonlinear patterns. Among the participants, 8 showed significant changes in daily step counts over time (all *P*<.05; see Table S5 in Multimedia Appendix 1 for the *P* values), with 7 showing improvements and 1 experiencing a decline. Visualizations of the daily step trajectories for these 8 participants are presented in Figure 5, with the complete set of plots available in Figure S1 in Multimedia Appendix 1.

### Secondary Outcomes

**Figure 6.**
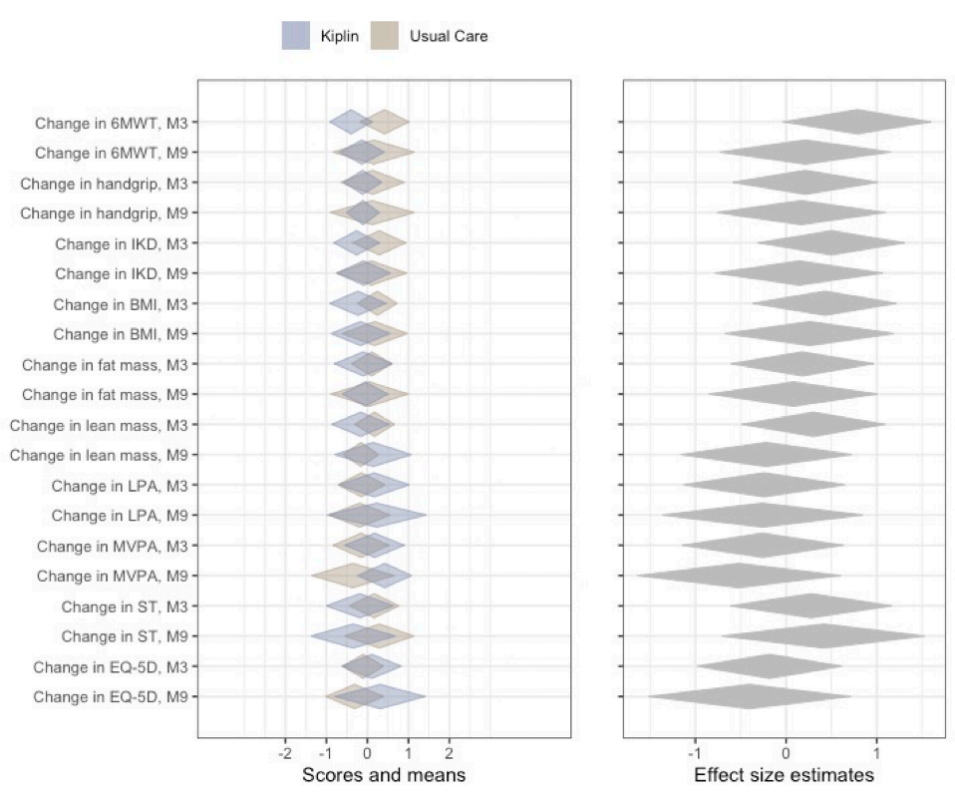
Diamond comparison plot of the univariate standardized changes in the secondary outcomes between the Kiplin and usual care conditions, with the middle of the diamonds showing the means and the endpoints of the diamonds representing the 99% CIs. 6MWT: 6-minute walk test; IKD: isokinetic dynamometer; LPA: light physical activity; M3: 3-month follow-up; M9: 9-month follow-up; MVPA: moderate-to-vigorous physical activity; ST: sedentary time.

Table 2 presents the results of the secondary outcome measures. Within-group comparisons showed significant improvements from baseline to 9 months in lower limb muscle strength (*ß*=6.68, 95% CI 1.40 to 11.96; *P*=.01) and a significant decrease in BMI at 3 months (*ß*=−0.51, 95% CI −0.98 to −0.04; *P*=.03) for the Kiplin group. In contrast, the control group showed significant improvements in quality of life at 3 months (*ß*=0.08, 95% CI 0.01 to 0.15; *P*=.02) and in 6MWT and lower limb muscle strength at both 3 months (*ß*=38.94, 95% CI 18.96 to 58.93; *P<*.001, and *ß*=9.27, 95% CI 4.15 to 14.40; *P*=.001, respectively) and 9 months (*ß*=25.41, 95% CI 2.04 to 48.78; *P*=.03, and *ß*=7.59, 95% CI 1.80 to 13.39; *P*=.01, respectively). Results of the mixed-effect models revealed no significant group × time interactions for secondary outcomes, except for a significant interaction in MVPA change from baseline favoring the Kiplin group (*ß*=5.69, 95% CI 0.61 to 10.77; *P*=.03). Figure 6 provides a visual comparison of changes between conditions with effect size estimates. Graphs depicting the evolution of secondary outcomes are available in Figure S2 in Multimedia Appendix 1.

**Table 2.** Secondary outcome measures at baseline, the 3-month follow-up, and the 9-month follow-up.

Outcome	Baseline, mean (SD)		3-month follow-up, mean (SD)		9-month follow-up, mean (SD)		Group-by-time interaction	
	Kiplin	Usual care	Kiplin	Usual care	Kiplin	Usual care	*ß* (95% CI)	*P* value
LPA^a^ (minutes/week)	81.38 (26.50)	90.65 (31.11)	87.47 (25.43)	88.87 (32.36)	88.12 (34.25)	85.98 (31.61)	5.29 (−4.01 to 14.59)	.26
MVPA^b^ (minutes/week)	27.91 (13.55)	32.88 (25.72)	32.60 (18.66)	27.40 (19.78)	36.14 (21.28)	26.06 (15.18)	5.69 (0.61 to 10.77)	.03
Sedentary time (minutes/week)	490.71 (32.81)	476.47 (50.33)	479.93 (35.07)	483.73 (48.58)	475.74 (44.35)	487.97 (43.67)	−11.05 (−23.18 to 1.07)	.07
6MWT^c^ (meters)	514.92 (66.02)	477.11 (77.80)	525.64 (69.88)	516.06 (93.93)	511.94 (71.01)	523.33 (63.34)	−15.03 (−32.19 to 2.12)	.09
Handgrip (kg)	35.09 (9.23)	32.54 (8.81)	35.29 (10.22)	33.74 (10.28)	36.12 (9.89)	35.41 (11.45)	−0.25 (−1.25 to 0.75)	.62
Lower limb muscle strength (kg)	53.13 (16.70)	49.76 (16.45)	57.22 (16.11)	59.03 (19.91)	60.65 (19.86)	58.21 (14.30)	−0.90 (−4.84 to 3.05)	.65
BMI (kg/m²)	39.7 (7.37)	40.3 (7.02)	39.5 (7.80)	40.4 (6.99)	41.5 (8.07)	40.6 (6.61)	−0.33 (−0.72; to 0.06)	.10
Fat mass (%)	44.7 (10.0)	45.8 (9.53)	44.9 (10.5)	45.6 (9.69)	47.7 (9.19)	46.4 (7.91)	−0.23 (−1.17 to 0.72)	.64
Lean mass (kg)	58.2 (13.4)	56.9 (10.6)	57.4 (12.5)	57.3 (10.9)	57.9 (12.0)	57.3 (11.1)	0.56 (−0.91 to 2.04)	.45
EQ-5D (index)	0.63 (0.17)	0.61 (0.19)	0.68 (0.18)	0.69 (0.10)	0.68 (0.16)	0.63 (0.19)	0.01 (−0.04 to 0.07)	.64

a LPA: light physical activity.

b MVPA: moderate-to-vigorous physical activity.

c 6MWT: 6-minute walk test.

### Economic Evaluation

The cost per patient in the usual care group was €491.92 (US $566.80) compared with €568.74 (US $655.31) in the Kiplin group, resulting in a cost differential (surplus of Kiplin compared with the control group) of €76.82 (US $88.51). Quality-adjusted life years (QALYs) did not significantly differ between groups and time points. The QALYs calculated from the EQ-5D questionnaire were, on average, rather good at baseline, with averages of 0.62 at T0 and 0.67 at T1 and T2.

## Discussion

### Principal Findings

The results of this RCT revealed that a group-based gamified digital intervention significantly increased daily PA compared with a traditional supervised, face-to-face APA program. Interestingly, this significant difference was observed both during the 3-month program (+1085 daily steps) and during follow-up periods (ie, 6 months postintervention: +1775 daily steps). These results remained consistent irrespective of adjustments for potential confounding factors, reinforcing the robustness of the findings.

In terms of trajectory, participants in the usual care group followed a U-shaped trajectory: their daily steps declined during the intervention and then returned to baseline by the program’s end, which is consistent with our hypothesis and the compensation mechanisms often observed between supervised and leisure-time PA in APA programs [27]. In contrast, Kiplin participants exhibited an inverted U-shaped pattern, with a sharp early surge in steps that gradually decelerated over time, suggesting the intervention was most potent at the outset.

### Comparison With Existing Literature

Our findings are consistent with previous research showing that gamified digital interventions can lead to meaningful real-world improvements in daily step counts, with effects lasting several months after the intervention [14,30,45]. We observed that the intervention’s impact was strongest at the beginning of the program, aligning with evidence that shorter gamified interventions tend to produce the largest gains [14,30]. Although the follow-up period in our study was longer than the average duration reported in previous research, the effect size 6 months postintervention (834 additional daily steps on average) observed in this study was greater than that of earlier trials [14].

To the best of our knowledge, no gamified digital program has previously been compared with usual care in the management of obesity and T2D. However, a prior meta-analysis comparing digital health interventions with minimal intervention or usual care in the context of cardiac rehabilitation found no significant effect on objectively measured PA [46]. In contrast, our study demonstrated that the digital intervention promoted greater increases in both daily step counts and MVPA compared with usual care. This effectiveness for MVPA is also novel, as previous meta-analyses did not report significant effects of gamified interventions on such outcomes [14].

### Clinical and Methodological Implications

These results are particularly promising for several reasons. Where current traditional face-to-face, supervised APA programs often face challenges for driving sustained increases in PA [26], the Kiplin program addresses this gap effectively. Notably, the effect size observed during the intervention was maintained throughout the 6-month follow-up, underscoring the sustainability of the PA behavior change. The benefits of improving daily PA are now well-recognized [47]. A recent meta-analysis revealed that taking more steps per day was associated with a progressively lower risk of all-cause mortality, regardless of age, health status, or intensity [48]. This suggests that the observed behavioral changes in the Kiplin group could have significant long-term health implications.

Nevertheless, in parallel to these results, idiographic analyses revealed substantial variability in individual responses within the Kiplin condition. More especially, GAM models showed (1) significant between- and within-person variability during the intervention, with some participants displaying highly nonlinear patterns while others showed linear trends, and (2) divergent responses to the intervention, with several participants experiencing no significant changes across time. These findings introduce important nuances to our results, indicating that digital interventions may not be suitable for every individual. This underscores the need for effective screening methods to identify patients who are most likely to benefit. Indeed, we can assume that factors such as the stage of behavior change, the acceptability of the technologies [49], or some physiological characteristics [50] could play a critical role in determining whether a digital or in-person program would be more appropriate.

In addition, no statistical differences were observed in the secondary outcomes evaluated in this study, except for the MVPA change. Although these results should be interpreted with caution, as they are exploratory and stem from secondary analyses potentially without appropriate statistical power, they illustrate 2 different approaches to chronic disease management: behavior change facilitated through the Kiplin program versus functional and physical fitness improvement achieved through the traditional program.

According to the cost-utility analysis, the cost surplus of approximately €76 per patient generated by Kiplin is not significant for an unimpaired quality of life. The anticipated gains in QALYS could not be demonstrated, probably due to the limited sample size at T2 and the high baseline quality of life score reported by patients at T0. This suggests that the generic EQ-5D measurement tool, while useful for QALY transposability, may lack the specificity needed to detect quality of life changes in this population of patients. To provide a conclusive evaluation of Kiplin’s cost-effectiveness over usual care, future research should conduct more specific medical-economic analyses, incorporating quality of life metrics adapted to the patient population. Although digital interventions are often expected to be more cost-effective than in-person programs, the intervention in this study proved to be more expensive. The higher costs observed in the Kiplin group primarily reflect the fixed expenses associated with implementing a hybrid digital intervention, including software licensing, human resources, and equipment. Given the relatively small number of participants in our study, these fixed costs could not be offset through economies of scale. In a larger-scale implementation, the average cost per participant would likely decrease substantially, making the digital component more cost-efficient in practice. Nevertheless, although unexpected, our findings are consistent with previous studies that have not demonstrated clear evidence of cost-effectiveness [20,51] .

From a methodological perspective, the variability observed with the GAMs reflects a broader limitation of traditional RCTs, which typically focus on group-level differences, potentially overlooking meaningful interindividual variability. By relying solely on aggregate measures, RCTs may fail to capture distinct response patterns, limiting insights into underlying mechanisms and moderating factors that influence intervention effectiveness. In this line, idiographic approaches appear to be a valuable complement to traditional clinical experimental designs, and this study highlights the importance of combining both between- and within-person approaches for evaluating digital interventions, as well as the advantage of high-resolution behavior measurement.

### Limitations and Perspectives

However, the results should be interpreted in light of several limitations. First, although using wearables allowed for continuous daily step monitoring in real-world conditions, variations in wear time between groups at the day level cannot be excluded, as the Kiplin group may have been more incentivized to wear the device through gamification. Qualitative interviews (not reported in this paper) with the patients suggested that participants in both groups wore the devices consistently (since recharging was not required), but we lack objective data to confirm this. In addition, these activity monitors are not able to assess several forms of PA, such as cycling or ergometer exercise. Future studies using wearables with continuous heart rate tracking could better assess and control for wear time, and devices such as Motus or SENS may allow for large-scale, 24-hour movement behavior assessment [52], including cycling [53]. Second, results from secondary outcomes should be viewed with caution due to potentially insufficient power to detect these effects as the power test was computed for the primary outcome according to a large effect size. Third, we observed baseline differences in daily steps between the 2 conditions, which were driven by a few participants with unusually high activity for people with obesity or T2D, as our sample reflected real-world hospital admissions without activity-based selection criteria. Future studies could screen for initial PA to minimize such variability. Last, digital interventions consist of a complex interplay of interconnected components, making it challenging to isolate the specific influence of individual elements when the intervention is evaluated as a whole. To address this challenge, innovative research frameworks like the Multiphase Optimization Strategy (MOST) [54] or hybrid designs [55] could offer a promising avenue in future research for systematically isolating the intervention components and optimizing digital health interventions.

### Conclusion

This study confirms the potential of digital health interventions to promote sustained changes in PA compared with usual care in participants with obesity and T2D. Although this behavior change has not led to superior clinical outcomes compared with usual care in this study, its continued persistence beyond the 6-month postintervention period could lead to more pronounced long-term benefits. To validate this hypothesis, larger clinical trials with extended follow-up durations are necessary. This study also had the originality of incorporating both between-group and within-person analyses of daily step counts. The findings indicate that, although the digital intervention effectively increased daily steps on average compared with usual care, the benefits were not uniform across all participants. This underscores the importance of patient screening and tailoring program content to individual needs. Future research should further investigate these considerations to optimize digital health intervention design and implementation.

## Supplementary material

10.2196/78376 Multimedia Appendix 1 Additional information about the study participants and details of the analyses, including visualizations and graphs.

10.2196/78376 Checklist 1 CONSORT checklist.

## Abbreviations

1RM: 1-repetition maximum
6MWT: 6-minute walk test
AIC: Akaike information criterion
APA: adapted physical activity
BIC: Bayesian information criterion
CONSORT: Consolidated Standards of Reporting Trials
DIPPAO: Digital Intervention Promoting Physical Activity among Obese people
edf: effective degrees of freedom
GAM: generalized additive model
LPA: light physical activity
MOST: Multiphase Optimization Strategy
MVPA: moderate-to-vigorous physical activity
PA: physical activity
QALYs: quality-adjusted life years
RCT: randomized controlled trial
T2D: type 2 diabetes
