# Supplementary material for: Effect of a Digital Health Physical Activity Program Integrating Gamification for Obesity Management in Comparison With Usual Care: Randomized Controlled Trial With an Ideographic Approach
Source: J Med Internet Res. 2025 Nov 28;27:e78376. doi: 10.2196/78376 (PMC12669915; doi:10.2196/78376)
Supplement: Multimedia Appendix 1 [file jmir-v27-e78376-s001.pdf]

Effect of a Digital Health Physical Activity Program Integrating Gamification for Obesity Management in Comparison With Usual Care: Randomized Controlled Trial With an Ideographic Approach.

Alexandre Mazéas, Aïna Chalabaev, Marine Blond, Charline Mourgues, Bruno Peirera, Martine Duclos

Supplementary Materials

1. Inclusion and exclusion criteria of the study
2. Baseline characteristics of randomized participants (ITT sample)
3. Unadjusted and adjusted models for the primary outcome analyses
4. Results of the Generative Additive Models
5. Visualization of individual time series analyses (GAMs) for patients of the Kiplin group
6. Graphs depicting the evolution of secondary outcomes for each condition

All Data and materials are available on the OSF page of the project (DOI 10.17605/OSF.IO/YPD5S)

Correspondence: Dr Alexandre Mazéas; amazeas@health.sdu.dk

1. Inclusion and exclusion criteria of the study

Table S1. Inclusion and exclusion criteria.

Inclusion criteria	• Voluntary patients affected by obesity (BMI ≥30 kg/m2 and <45 kg/m2) and/or overweight/ obesity and T2DM, aged 18–65 years, male or female, and referred to the department of sports medicine of the University Hospital of Clermont-Ferrand by their physician to benefit from supervised physical activity.<br>• Possession of a smartphone with a compatible operating system (at least iOS12 or Android 6.0).<br>• Subject covered by health social security.<br>• Subject naive to any APA intervention.
Exclusion criteria	• Medical or surgical history judged by the investigator to be incompatible with the study.<br>• Subject with an unstable psychiatric condition.<br>• Pregnant or breastfeeding women.<br>• Heavy alcohol consumption (> 2 to 3 drinks per day depending on gender) or drug addiction.<br>• Disability or contraindication to PA.<br>• Subject with cardiorespiratory and/or osteoarticular disorders that limit their ability to perform physical tests or moderate physical activity for 30 minutes.<br>• Subject with progressive cardiovascular or neoplastic disease.<br>• Subject who has presented a major infection in the 3 months prior to inclusion.<br>• Subject with a known neuro-muscular pathology (i.e., myopathy, myasthenia, rhabdomyolysis, paraplegia, hemiplegia).<br>• Subject with chronic or acute inflammatory pathology within 3 months prior to inclusion.<br>• Subject diagnosed and/or treated for schizophrenia, bipolar disorder, major depression.<br>• Subject deprived of their liberty by judicial or administrative decision.<br>• Subject refusing to sign the written consent to participate.<br>• Subject participating in another study.

2. Baseline characteristics of randomized participants (ITT sample)

Table S2. Descriptive statistics of the intention to treat sample.

	**Kiplin intervention** (n = 25)	**Usual care** (n = 25)	**Total** (n = 50)
Sociodemographics			
Age, mean (SD)	47.46 (11.18)	48.62 (14.35)	47.90 (12.49)
Female, n (%)	17 (68)	20 (80)	37 (74)
BMI kg/m², mean (SD)	39.89 (7.32)	40.76 (7.43)	40.22 (7.38)
Obese, n (%)	22 (88)	24 (96)	46 (92)
T2D, n (%)	7 (28)	8 (32)	15 (30)
Education			
Less than high school, n (%)	7 (28)	9 (36)	16 (32)
High school, n (%)	6 (24)	6 (24)	12 (24)
University degree (%)	12 (48)	10 (40)	22 (44)
Physical activity (daily steps)			
Baseline, mean (SD)	6,248 (2,536.92)	6,826 (3,232.71)	6,494 (2,828.74)

3. Unadjusted and adjusted models for the primary outcome analyses

Table S3. Unadjusted and adjusted mixed-effect models for the primary outcome analyses predicting daily step count over time.

	Unadjusted model			Adjusted model		
	b [95 CI]	SE	*P*	*b* [95 CI]	SE	*P*
**Intercept**	7442.39 [5523.09; 9361.69]	978.94	**<0.001**	10967.49 [3625.79; 18309.19]	3744.36	**0.003**
**Fixed Effects**						
Time	-20.06 [-41.82; 1.69]	11.09	0.071	-23.96 [-61.33; 13.40]	19.05	0.209
Time^2	0.13 [-0.17; 0.09]	0.06	0.**030**	0.14 [-0.02; 0.31]	0.08	0.084
Condition						
Usual care	Reference			Reference		
Kiplin	-1464.86 [-4022.46; 1092.74]	1304.51	0.262	-767.51 [-5116.74; 3581.73]	2218.16	0.729
Participants' profile						
Age	-			-46.01 [-102.90; 10.87]	29.01	0.113
BMI	-			-90.17 [-185.63; 5.30]	48.69	0.064
Interaction time x condition						
Time * Kiplin	41.98 [13.59; 70.37]	14.48	**0.004**	47.91 [3.68; 92.14]	22.56	**0.034**
Time^2 * Kiplin	-0.23 [-0.39; -0.07]	0.08	**0.005**	-0.26 [-0.46; -0.05]	0.10	**0.013**
Season						
Season	-			-191.77 [-371.78; -11.76]	91.81	**0.037**
APA sessions						
Sessions performed	-			11.44 [-133.18; 156.06]	73.76	0.877
Baseline daily steps						
<5000	-			Reference		
5001-7500	-			1631.29 [54.33; 3208.25]	804.27	**0.043**
7501-10000	-			4356.72 [2622.59; 6090.85]	884.43	**<0.001**
>10000	-			8089.45 [5672.00; 10506.90]	1232.93	**<0.001**
log-Likelihood	-36131.878			-28857.528		
Akaike Information Criteria	72283.8			57749.1		
Marginal R^2 / Conditional R^2	0.003 / 0.620			0.351 / 0.665		

Table S4. Unadjusted and adjusted mixed-effect models for the primary outcome analyses predicting daily step count from period.

	**Unadjusted model**			**Adjusted model**		
	b [95 CI]	SE	*P*	*b* [95 CI]	SE	*P*
**Intercept**	6795.68 [5007.00; 8584.36]	912.45	**<0.001**	-1693.19 [-12274.87; 8888.58]	5397.78	0.754
**Fixed Effects**						
Phases of the study						
Baseline	Reference			Reference		
Intervention	7.52 [-441.36; 456.39]	228.98	0.974	-82.67 [-640.66; 475.33]	284.63	0.771
Follow-up	-940.54 [-1652.09; -229.00]	362.97	**0.010**	-1638.60 [-2645.62; -631.58]	513.68	**0.001**
Participants' profile						
Age	-			51.38 [-34.74; 137.50]	43.93	0.242
BMI	-			-112.33 [-246.06; 21.40]	68.22	0.100
Condition						
Usual care	Reference			Reference		
Kiplin	-712.02 [-3144.54; 1720.49]	1240.88	0.556	5171.96 [1102.21; 9241.72]	2075.99	**0.013**
Season						
Season	-			49.91 [-38.07; 137.88]	44.88	0.266
APA sessions						
Sessions performed	-			186.19 [-48.65; 421.04]	119.80	0.120
Baseline daily steps						
<5000	-			Reference		
5001-7500	-			3831.62 [1632.67; 6030.57]	1121.69	**0.01**
7501-10000	-			3420.70 [610.98; 6930.41]	1433.24	**0.017**
>10000	-			6414.01 [2941.36; 9886.66]	1771.41	**<0.001**
Interaction phase x group						
Kiplin x intervention	1084.15 [493.43; 1675.65]	301.54	**<.001**	1241.35 [569.53; 1913.17]	342.70	**<0.001**
Kiplin x follow-up	1774.76 [908.41; 2720.35]	469.46	**<.001**	2550.29 [1419.71; 3680.87]	576.71	**<0.001**
-2*log (lh)	-64001.5			-51923.5		
Akaike Information Criteria	128023.1			120178.5		
Marginal R^2 / Conditional R^2	0.006 / 0.644			0.255 / 0.804		

4. Results of the Generative Additive Models

Table S5. Results of the Generative Additive Models evaluating the evolution of daily steps across time between baseline and one week after the end of the intervention for participants of the Kiplin group.

	Estimate	edf	*P*
Participant #1	1.27	4.51	0.31
Participant #2	3.86	1.94	**0.01**
Participant #3	0.94	1.00	0.33
Participant #4	0.32	1.00	0.58
Participant #5	1.39	1.27	0.34
Participant #6	0.44	1.53	0.57
Participant #7	3.06	8.10	**<.001**
Participant #8	4.50	5.75	**<.001**
Participant #9	2.76	4.56	**0.02**
Participant #10	0.01	1.00	0.92
Participant #11	2.49	2.00	0.09
Participant #12	13.14	1.25	**<.001**
Participant #13	2.07	4.54	0.07
Participant #14	2.83	1.00	0.10
Participant #15	7.61	5.54	**<.001**
Participant #16	0.05	1.00	0.83
Participant #17	1.28	2.32	0.31
Participant #18	1.64	5.93	0.15
Participant #19	8.62	1.29	**<.001**
Participant #20	1.60	5.82	0.15
Participant #21	3.17	7.81	**<.001**

5. Visualization of individual time series analyses and GAMs for patients of the Kiplin group

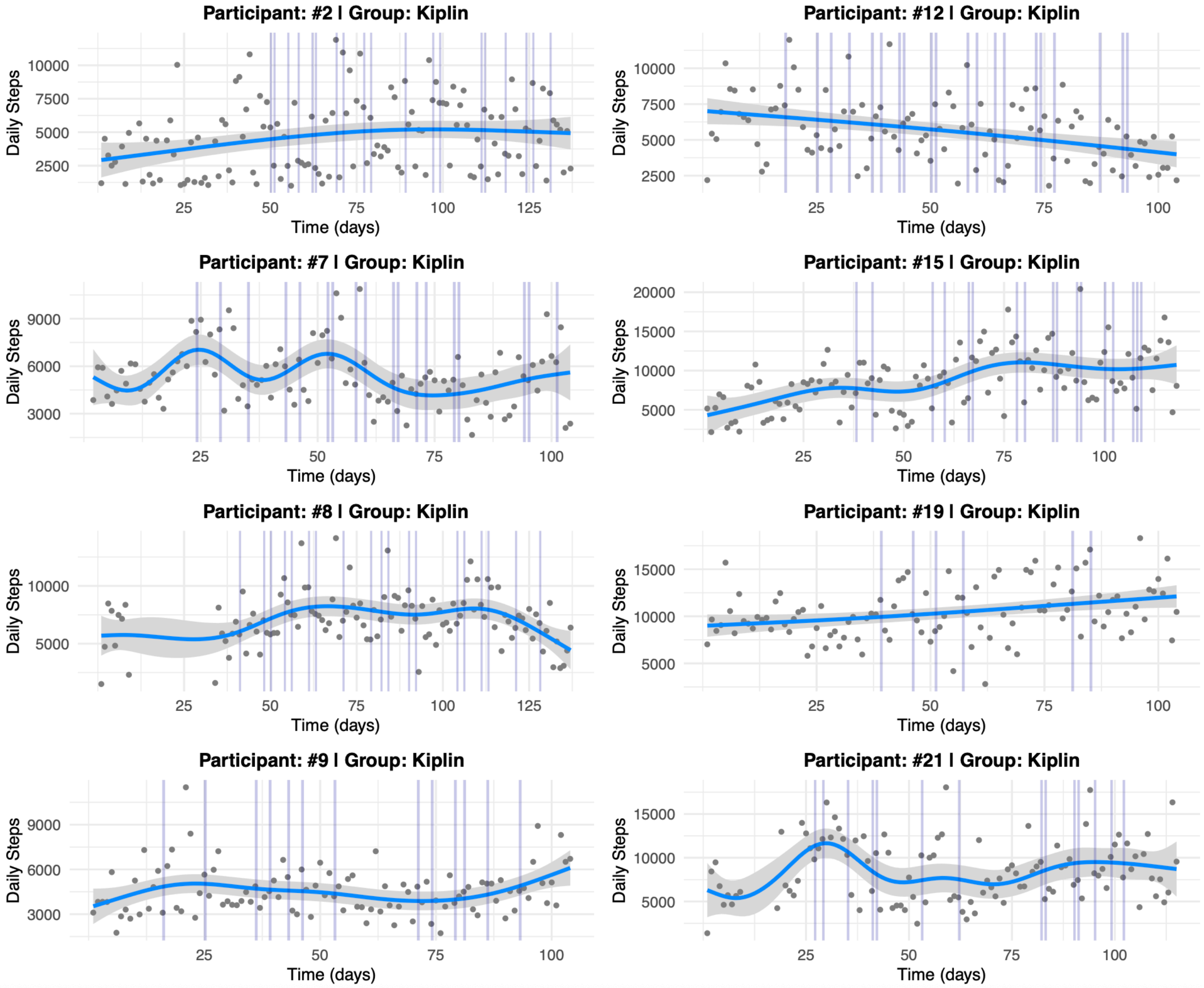

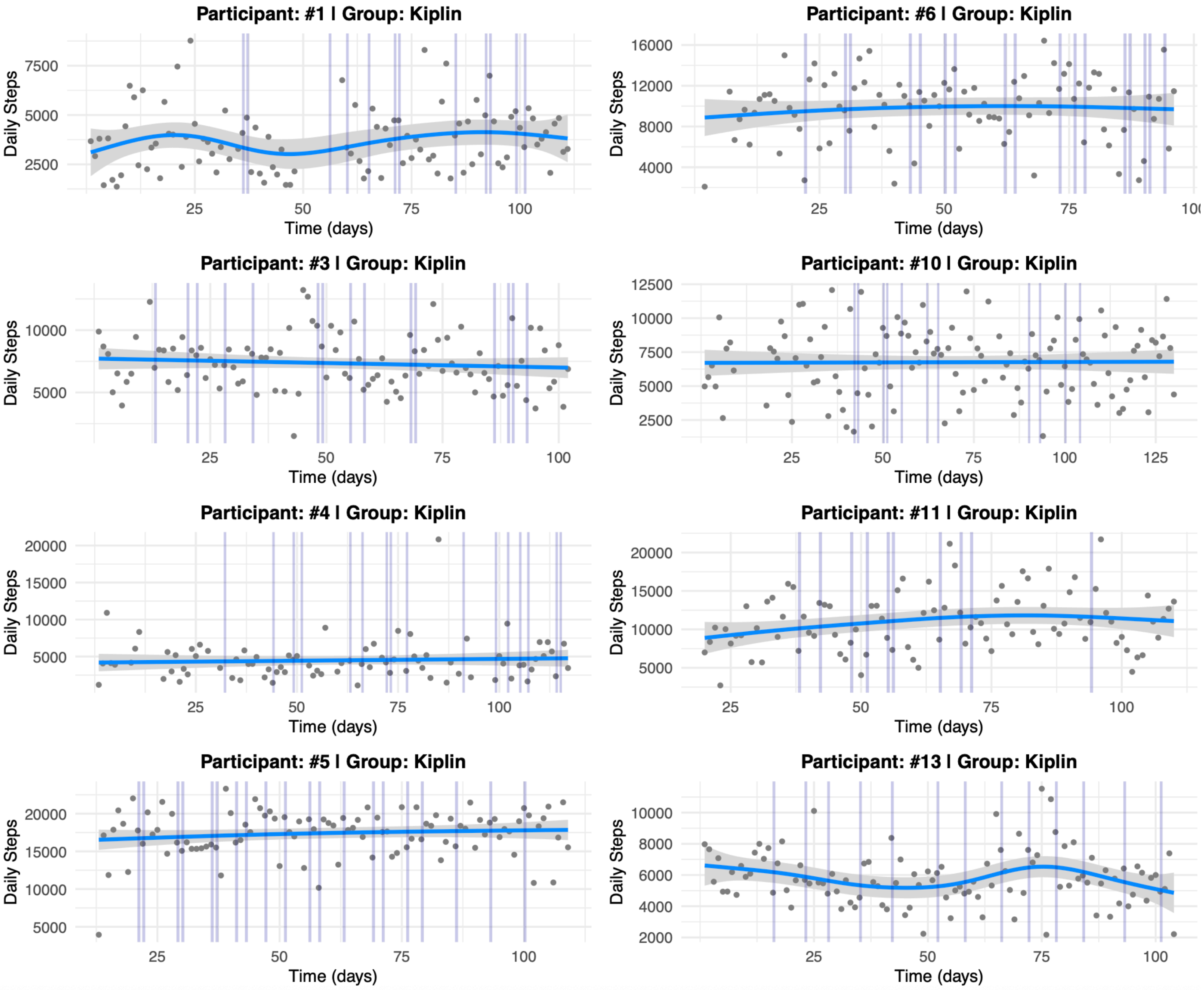
Participant: #1 | Group: Kiplin
Daily Steps
Time (days)
Participant: #6 | Group: Kiplin
Daily Steps
Time (days)
Participant: #3 | Group: Kiplin
Daily Steps
Time (days)
Participant: #10 | Group: Kiplin
Daily Steps
Time (days)
Participant: #4 | Group: Kiplin
Daily Steps
Time (days)
Participant: #11 | Group: Kiplin
Daily Steps
Time (days)
Participant: #5 | Group: Kiplin
Daily Steps
Time (days)
Participant: #13 | Group: Kiplin
Daily Steps
Time (days)

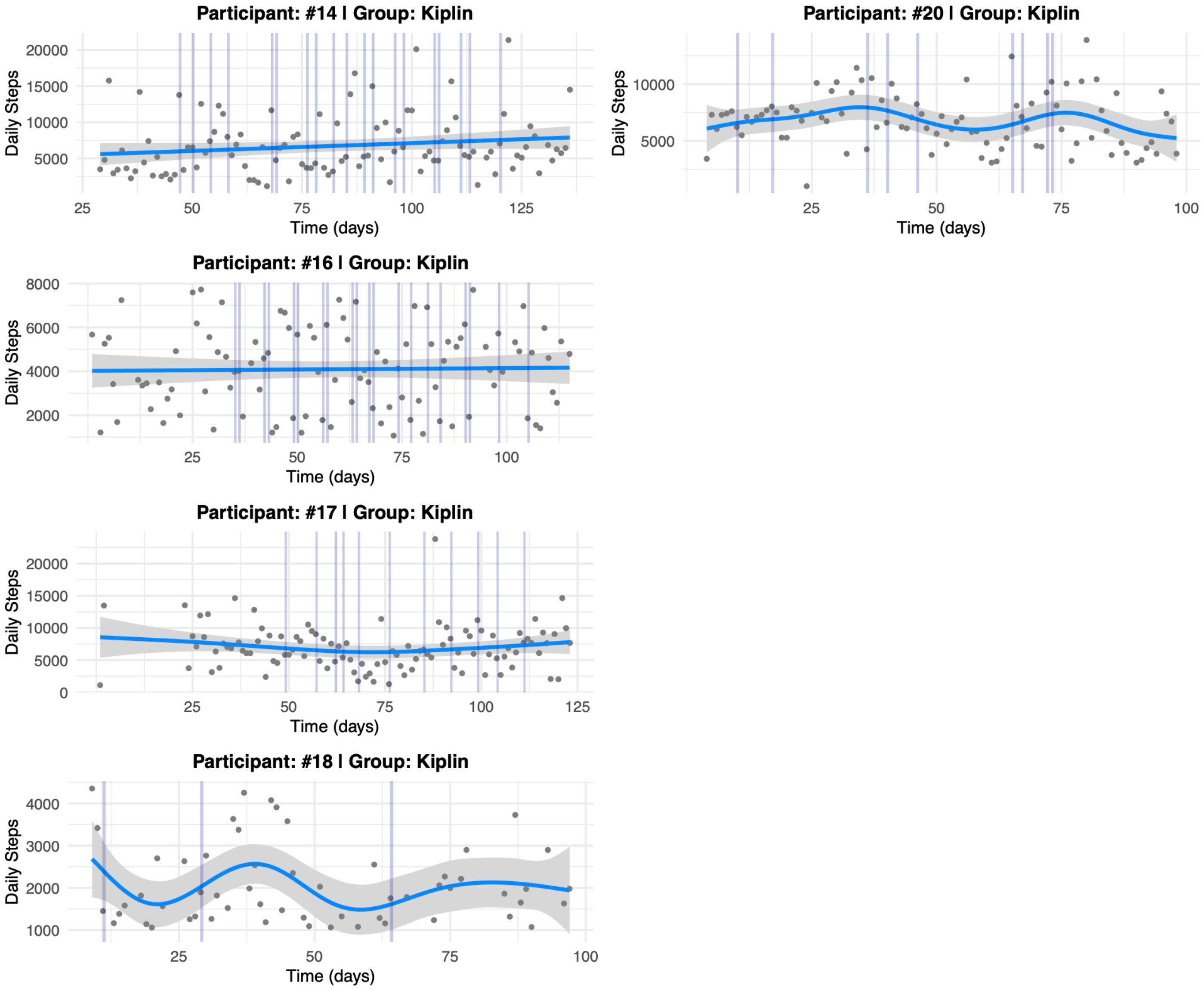

Figure S1. Plots of the Generalized Additive Models for the evolution of daily steps from baseline to one-week post-intervention. Vertical lines represent APA sessions attended.

6. Graphs depicting the evolution of secondary outcomes for each condition

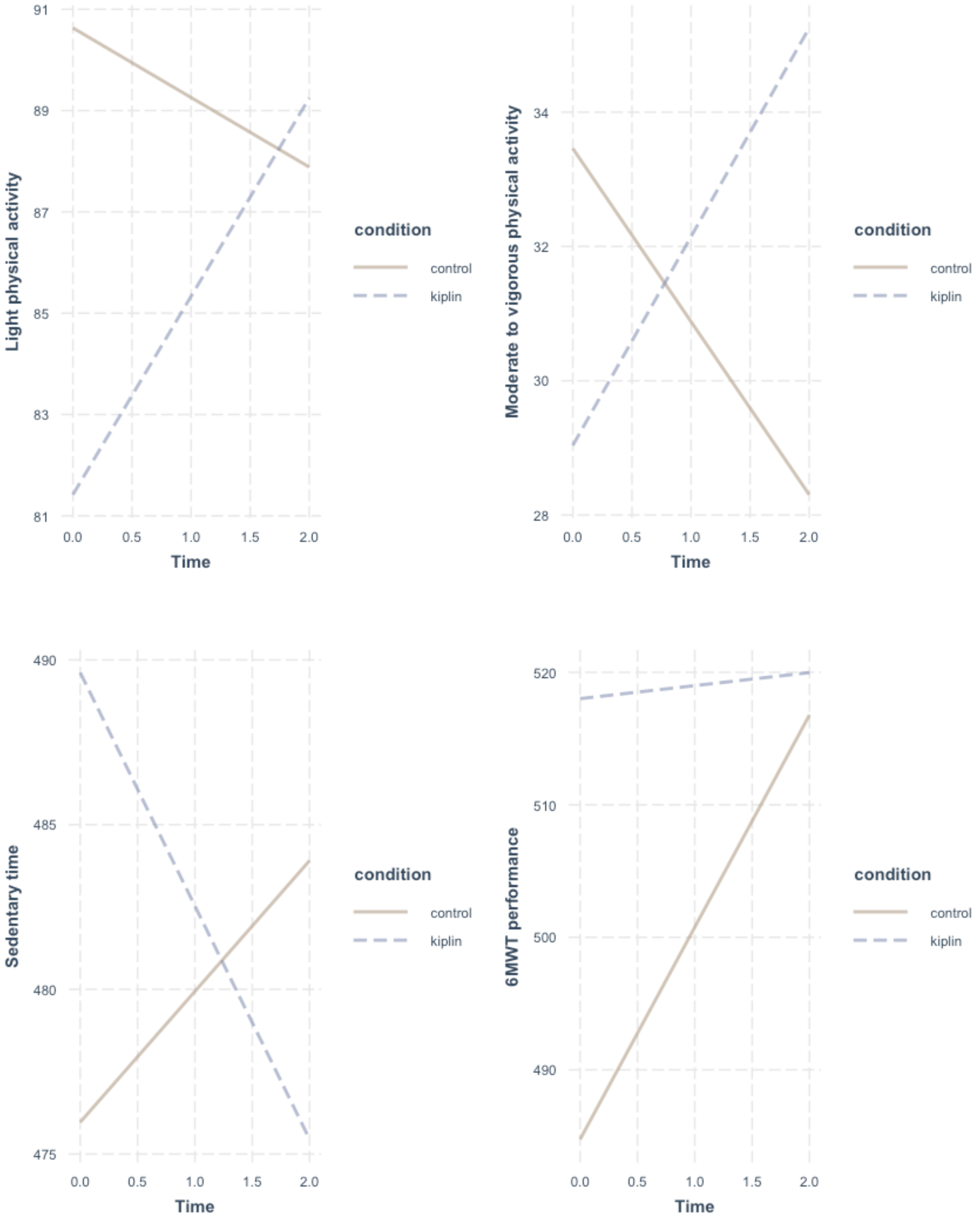

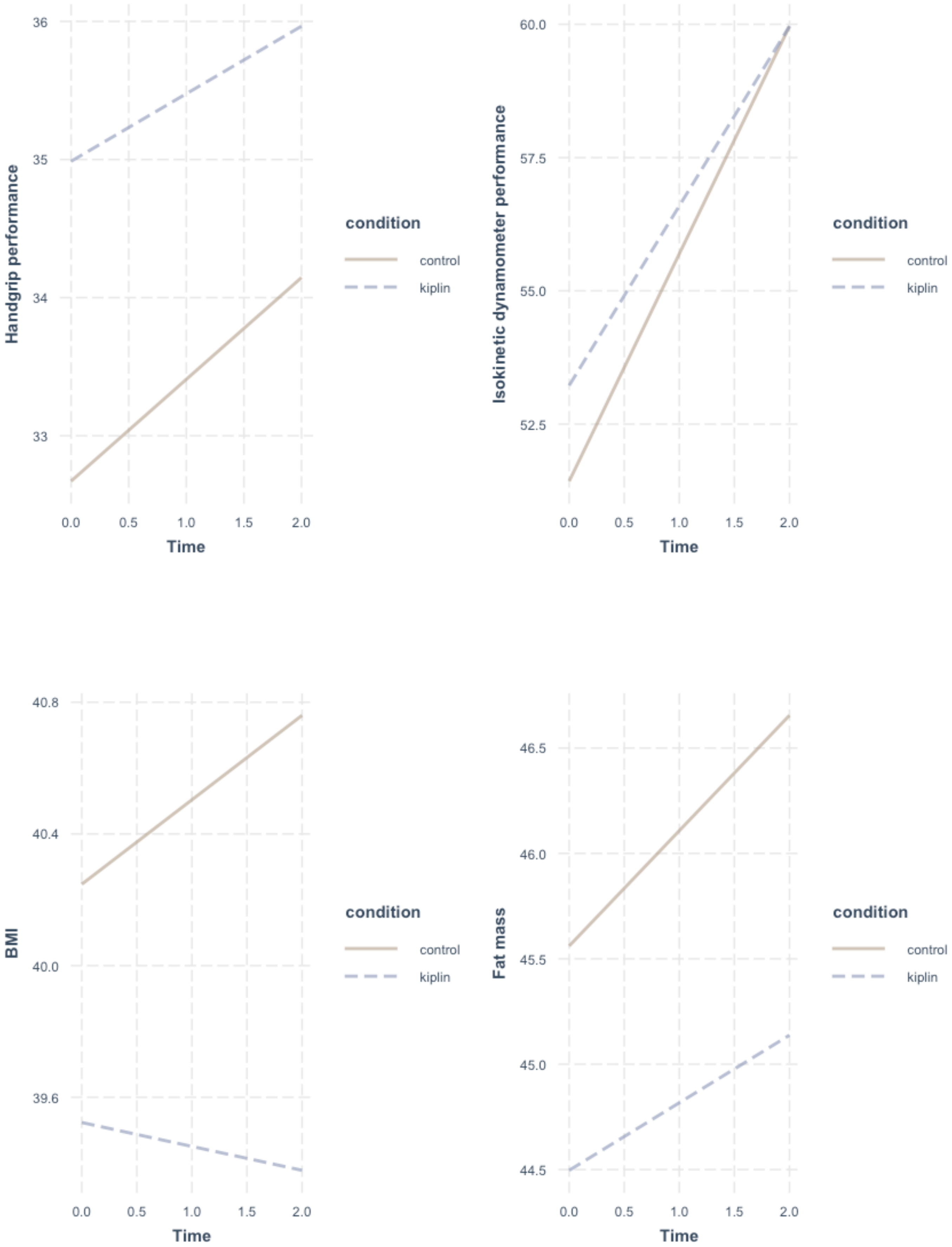
Handgrip performance
36
35
34
33
0.0
0.5
1.0
1.5
2.0
Time
condition
control
kiplin
Isokinetic dynamometer performance
60.0
57.5
55.0
52.5
0.0
0.5
1.0
1.5
2.0
Time
condition
control
kiplin
BMI
40.8
40.4
40.0
39.6
0.0
0.5
1.0
1.5
2.0
Time
condition
control
kiplin
Fat mass
46.5
46.0
45.5
45.0
44.5
0.0
0.5
1.0
1.5
2.0
Time
condition
control
kiplin

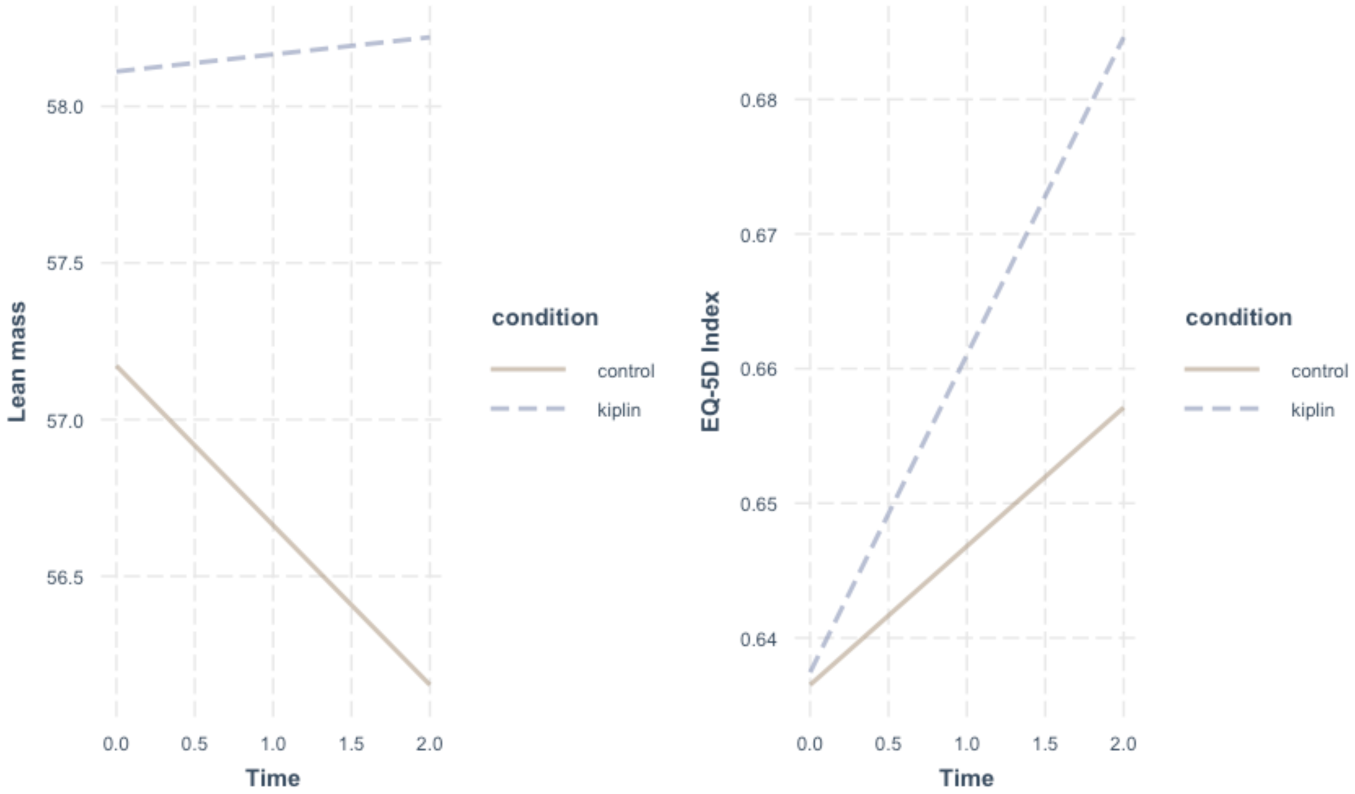

Figure S2. Graphs depicting the evolution of secondary outcomes for both conditions.
